# Highly intense monocycle terahertz vortex generation by utilizing a Tsurupica spiral phase plate

**DOI:** 10.1038/srep38880

**Published:** 2016-12-14

**Authors:** Katsuhiko Miyamoto, Bong Joo Kang, Won Tae Kim, Yuta Sasaki, Hiromasa Niinomi, Koji Suizu, Fabian Rotermund, Takashige Omatsu

**Affiliations:** 1 Graduate School of Advanced Integration Science, Chiba University, 1-33, Yayoi-cho, Inage-ku, Chiba 263-8522, Japan; 2 Molecular Chirality Research Center, Chiba University, 1-33, Yayoi-cho, Inage-ku, Chiba 263-8522, Japan; 3 Department of Physics and Department of Energy Systems Research, Ajou University, 206 Worldcup-ro, Yeongtong-gu, Suwon 16499, Korea; 4 Department of Electrical, Electronics and Computer Engineering, Chiba Institute of Technology, 2-17-1 Tsudanuma, Narashino, Chiba 275-0016, Japan; 5 Department of Physics, Korea Advanced Institute of Science and Technology, 291 Daehak-ro, Yuseong-gu, Daejeon 34141, Korea

## Abstract

Optical vortex, possessing an annular intensity profile and an orbital angular momentum (characterized by an integer termed a topological charge) associated with a helical wavefront, has attracted great attention for diverse applications due to its unique properties. In particular for terahertz (THz) frequency range, several approaches for THz vortex generation, including molded phase plates consisting of metal slit antennas, achromatic polarization elements and binary-diffractive optical elements, have been recently proposed, however, they are typically designed for a specific frequency. Here, we demonstrate highly intense broadband monocycle vortex generation near 0.6 THz by utilizing a polymeric Tsurupica spiral phase plate in combination with tilted-pulse-front optical rectification in a prism-cut LiNbO_3_ crystal. A maximum peak power of 2.3 MW was obtained for THz vortex output with an expected topological charge of 1.15. Furthermore, we applied the highly intense THz vortex beam for studying unique nonlinear behaviors in bilayer graphene towards the development of nonlinear super-resolution THz microscopy and imaging system.

Terahertz imaging systems, which enable the assignment of various eigen frequencies (molecular fingerprints) of molecules and clusters^1^, have been intensively investigated in a variety of fields, such as biomedicine, security, and nondestructive inspection^2^,^3^,^4^,^5^,^6^,^7^. However, their spatial resolution is typically diffraction-limited to the submillimeter scale due to the relatively long wavelength of THz radiation.

One technique for achieving super-resolution, i.e., resolution beyond the diffraction limit, is to exploit fluorescence depletion arising from linear or nonlinear phenomena, such as stimulated emission and up-conversion excitation. Nanometer spatial resolution has been successfully demonstrated for visible and near-infrared microscopy using optical vortex pulses^8^,^9^,^10^.

Optical vortices^11^,^12^ have an annular intensity profile, an orbital angular momentum characterized by an integer, *ℓ* (called the topological charge), and a helicity defined by the sign of the topological charge. They have potential applications in a variety of research areas, such as optical trapping and manipulation^13^,^14^, terabit high-speed optical communications^15^,^16^, and chiral or achiral nanostructure fabrication^17^,^18^,^19^,^20^,^21^. Laguerre-Gaussian (LG) modes, which are eigen modes of the para-axial electromagnetic equation in cylindrical coordinates, are well known as conventional optical vortices.

Highly intense and monocycle THz vortex pulses generated by the optical rectification of femtosecond laser pulses enable the study of nonlinear phenomena, e.g., nonlinear absorption and multi-photon excitation, and have the potential to enable THz imaging with a spatial resolution of micrometers (i.e., beyond the diffraction limit), which would allow the observation of local defects in crystalline materials such as graphene^22^ and various semiconductors^23^,^24^,^25^,^26^.

Several optical devices, including molded phase plates consisting of V-shaped slit antennas^27^ on thin metal, achromatic polarization elements^28^, and binary-diffractive optical elements^29^, have been recently proposed for THz vortex generation; however, they are typically designed for a specific frequency, and their narrow spectral bandwidth and low transmission often limit the mode conversion efficiency from the incident beam to the vortex beam to a few percent.

In a previous study, we successfully demonstrated the generation of a monochromatic THz vortex beam with a topological charge of *ℓ* = ±1 or 2 by utilizing a Tsurupica spiral phase plate (Tsurupica-SPP)^30^. A mode conversion efficiency of over 50% was obtained in the frequency region of 2–4 THz. Due to their extremely low dispersion and high transmission in the THz region, Tsurupica-SPPs have the potential to create highly intense monocycle THz vortex pulses.

In this study, we demonstrate for the first time the generation of highly intense monocycle vortex pulses centered at 0.6 THz using a femtosecond-laser-based THz source in combination with a Tsurupica-SPP. In addition, unique saturable absorption behaviors in bilayer graphene were investigated by utilizing the THz vortex pumping method as a milestone toward the development of nonlinear super-resolution THz microscopy and imaging.

## Results

### THz vortex generation

**Figure 1:**
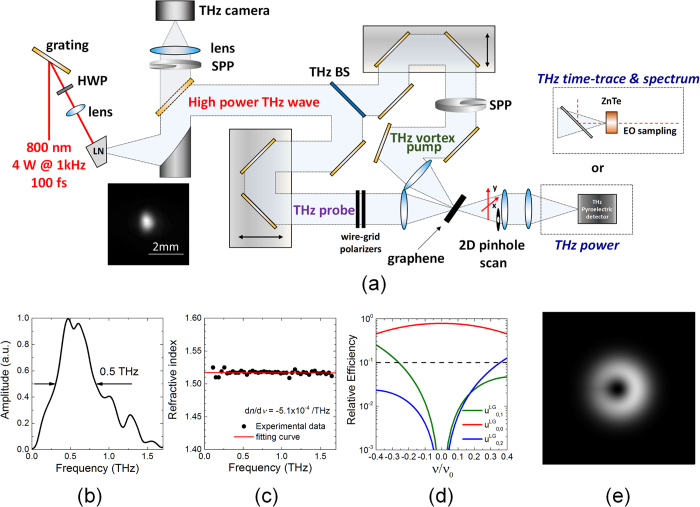
(**a**) Pinhole-scanning THz pump-probe system to measure nonlinear absorption of graphene. The pump and probe beams exhibited the vortex and Gaussian spatial forms, respectively. The spatio-temporal profile of the THz probe pulse was measured by employing an electro-optic sampling system and a THz pyroelectric detector. The inset showed the intensity profile of the THz Gaussian beam from the LiNbO_3_ crystal. (**b**) Experimental spectrum of the monocycle THz pulse. (**c**) Experimental refractive index of Tsurupica polymer measured by THz time domain spectroscopy. (**d**) Simulated relative intensity of the LG modes as a function of the frequency of the THz output (*v*_0_ = 0.6 THz). (**e**) Simulated near fields of the THz vortex output generated by utilizing a SPP.

A 1-kHz femtosecond Ti:sapphire regenerative amplifier system (Spitfire Ace, Spectra Physics; λ = 800 nm, 100-fs pulse duration, 4-W average power) with a tilted-pulse-front technique was used to pump a MgO-doped prism-cut stoichiometric LiNbO_3_ crystal for efficient optical rectification^31^(Fig. 1(a)). The generated THz output had a peak frequency of ~0.6 THz with a spectral bandwidth (FWHM) of 0.5 THz as shown in Fig. 1(b).

A Tsurupica-SPP (*φ*50.4-mm aperture, 2-mm thickness), designed to generate a first-order THz optical vortex with a topological charge of *ℓ* = 1 at a center frequency *v*_0_ of 0.6 THz, was employed. The SPP was azimuthally divided into 18 segments with a *nπ*/9 phase shift (where *n* is an integer between 0 and 17) with a spiral turn step of about 960 μm.

As related in our previous publication, the Tsurupica-SPP with high transmission and extremely low dispersion (*dn/dv* = −5.1 × 10^−4^/THz, Fig. 1(c)) has essentially the potential to convert the THz output with a relatively broad spectrum bandwidth, i.e., a bandwidth of at least 50% (0.3 THz) center frequency (0.6 THz), defined as the frequency range in which the relative intensities of undesired modes with *ℓ* = 0 and *ℓ* = 2 are less than 10%, to the vortex output without degradation of the beam quality (see Fig. 1(d)).

The average power of the vortex beam was measured using a calibrated pyroelectric detector (THZ5B-MT-DZ, Gentec-EO) to be 2.3 mW, corresponding to the peak power of 2.3 MW. The optical conversion efficiency of 65% from the THz beam to the vortex output was then obtained.

The THz vortex electric field *u*(*r*, *ϕ*) produce by the SPP is given by,


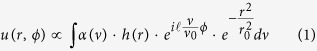



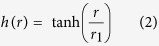


where *α*(*v*) is the amplitude spectrum function, *v* is the frequency of THz output, *r* is the radial coordinate, *ϕ* is the azimuthal coordinate, *h*(*r*) is the vortex core function^32^, *ℓ* is the topological charge of the SPP, and *r*_0_ and *r*_1_ are the mode-field and dark core radii. The resulting intensity *I*(*r*,*ϕ*) can be expressed as


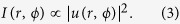


Substituting the experimental power spectrum and *r*_1_(=0.8*r*_0_) (see Fig. 1(b)) into Eq. (1), the spatial form of the THz beam can be simulated as shown in Fig. 1(e). The simulated THz beam exhibits a slightly off-center dark core along the -*x* direction.

In general, the THz vortex electric field *u*(*r*, *ϕ*) includes undesired higher-order LG modes *u*_*ℓ*,*p*_(*r*, *ϕ*) along the radial and azimuthal directions, and it can also be expressed as


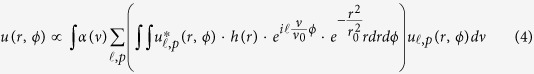



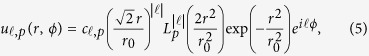


where *c*_ℓ,*p*_ is the normalization coefficient, *L*_*p*_^| ℓ |^ is the associated Laguerre polynomial, and *p* is the radial index.

Thus, the expected topological charge, <*l*>, of the simulated THz vortex beam is given by


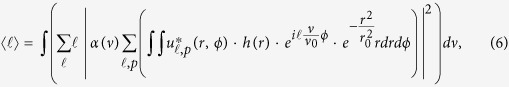


from which it is estimated to be +1.15.

The collimated THz Gaussian beam (30-mm beam diameter) was directed toward the SPP, where it is converted into the vortex beam. The focused THz vortex beam was then observed using a THz imager (NEC IRV-T0831; 23.5-μm spatial resolution) for analysis of its spatial profile. In addition, the average output power of the THz vortex beam was measured using the calibrated pyroelectric detector.

**Figure 2:**
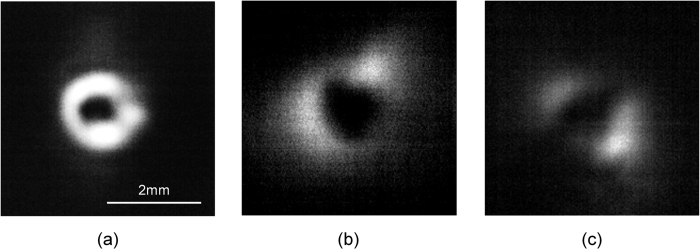
(**a**,**b**) Experimental near- and far- field of the THz vortex outputs. (**c**) THz vortex output produced by a tilted lens.

The generated vortex beam exhibited a *φ*2.3 mm annular mode with a *φ*400 μm off-center dark core (see Fig. 2(a)), and it is in good agreement with the theoretical simulations presented in Fig. 1(c). The off-center dark core also rotated approximately 90 degrees in the clockwise direction in the far-field, indicating that the topological charge was positive (see Fig. 2(b)).

The topological charge of the THz vortex beam was measured using an astigmatic focusing technique with a titled focusing lens. The cylindrical symmetry of the beam was further broken by astigmatic aberration of the lens^33^, which converted the vortex beam into a HG_01_-shaped beam (see Fig. 2(c)), indicating that the topological charge was approximately +1. Such tilted focusing lens method, in which the number of the dark fringes in the far-field is merely counted, is given by only one significant figure with a relatively large uncertainty. To assign quantitatively the topological charge of the vortex output, the orbital angular momentum analysis based on interferometric technique^34^,^35^, which is difficult to be performed in the THz region, will be needed.

Also, notice that the resulting THz vortex output shows a fully identical pulse width with that of the incident THz pulse because of the extremely low group velocity dispersion (~1fs^2^/mm) of the Tsurupica SPP.

### Nonlinear absorption in bilayer graphene

Graphene, which is a two-dimensional layer of carbon atoms arranged in a honeycomb lattice, exhibits a broadband absorption from terahertz to optical frequencies that originates from a conical electronic band around the Dirac point. In particular, it exhibits unique nonlinear absorption of terahertz waves owing to intraband scattering arising from the transient heating of electrons.

**Figure 3:**
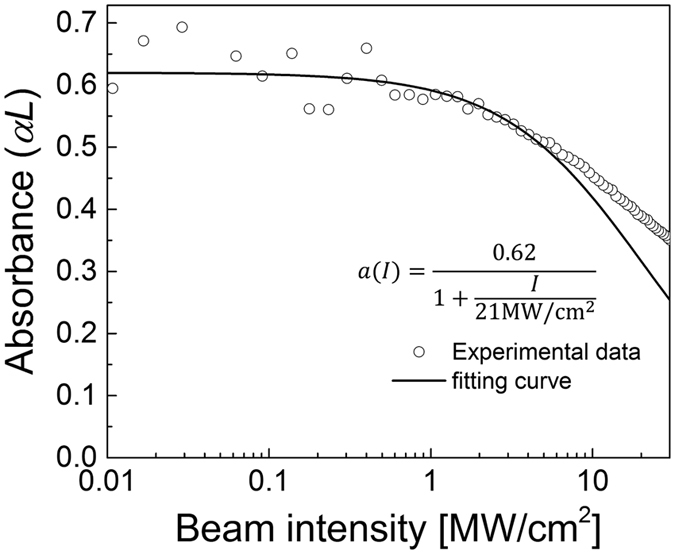
Experimental absorption plots of a bilayer graphene as a function of incident THz intensity. The solid line denotes a theoretical fit.

Bilayer graphene deposited on a quartz substrate was prepared, and its nonlinear absorption in the THz region was investigated using intensity-dependent transmission measurements with the THz vortex beam. As shown in Fig. 3, the saturation intensity *I*_*s*_, which is an important parameter for nonlinear absorption, and the linear absorption coefficient *a*_0_ were estimated to be ~21 MW/cm^2^ (the corresponding electric field of ~60 kV/cm) and 0.62, respectively, by fitting to the following absorption equation: *a*(*I*) = *a*_0_/(1 + *I*/*I*_*s*_).

When pumped by an intense THz vortex beam, the graphene should undergo unsaturated linear absorption only around the dark core of the vortex, thereby enabling super-resolution measurements of the linear absorption by utilizing a weak probe THz beam with a Gaussian profile.

Thus, the absorption of the graphene was further investigated by employing a degenerate pump-probe method, where a weak THz Gaussian beam and a strong THz vortex beam were used as probe and pump pulses, respectively. The pump and probe vortex pulses were temporally and spatially overlapped on the bilayer graphene sample. The intensity of the focused THz vortex on the bilayer graphene was then estimated to be 76 MW/cm^2^ (~3.7*I*_*s*_), corresponding to the electric field of ~234 kV/cm.

By using the saturable absorption function *a*(*I*), the transmitted probe beam (i.e., signal beam) can be simulated as follows:


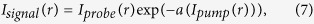


where *I*_*probe*_(*r*)(∝exp(−2*r*^2^/*r*_0_^2^)) is the incident THz probe beam, and *I*_*pump*_(*r*) is the THz pump beam (presented in Fig. 1(e)), respectively.

**Figure 4:**
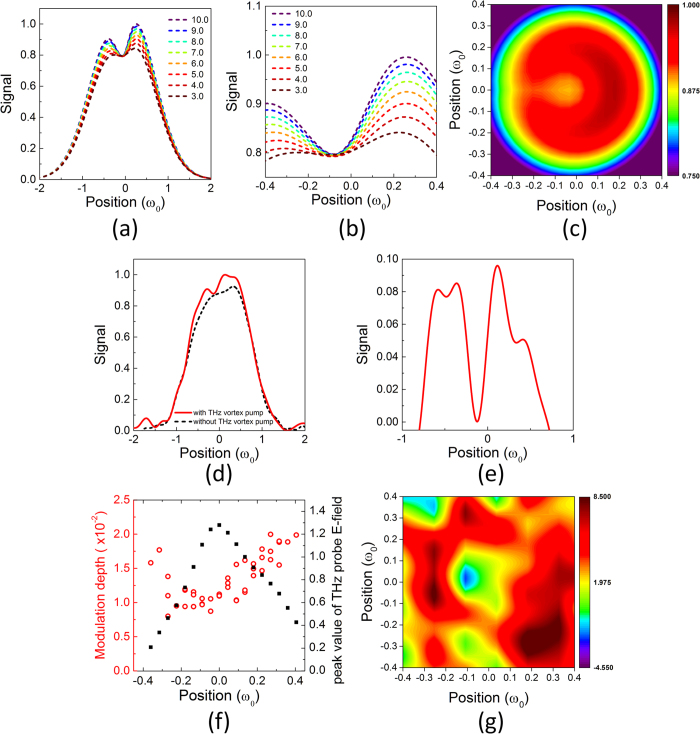
(**a**,**b**) Simulated spatial forms of the signal beam *I*_signal_(r) at various peak intensities of the vortex pump (3*I*_*s*_–10*I*_*s*_). (**c**) Simulated 2-dimentional spatial form of the vortex pump beam at a vortex pump peak intensity of 4*I*_*s*_. (**d**) Experimental spatial form of the signal beam with or without vortex pump beam (*ω*_0_ = 8 mm). (**e**) The linear absorption signal defined as the intensity difference between the signal beams with and without the vortex pump. (**f**) Experimental modulation depth of the signal electric field with or without vortex pump beam (*ω*_0_ = 11 mm). (**g**) Experimentally observed modulated 2-dimentinal spatial form with or without vortex pump beam (*ω*_0_ = 11 mm).

The simulated signal beam exhibits an off-axial shallow dip owing to the unsaturated linear absorption arising from the dark core of the vortex pump pulse (see Fig. 4(a–c)). The peak intensity of the vortex pump pulse was ranged within 3*I*_*s*_–10*I*_*s*_.

The spatial form of the signal beam was measured by scanning a pinhole based on pump-probe methods in the magnified image plane of the sample (magnification factor of ~8) with or without vortex pump beam. The signal beam then exhibited a slightly off-center shallow dip with a width of 3 mm (corresponding to the 1/7 of the diffraction limit) due to the strong linear absorption, whereas it exhibited a nearly Gaussian profile without the vortex pump pulse (Fig. 4(d)). In fact, the linear absorption signal (the relatively intensity depth ~8–9%), determined by intensity difference between the signal beams with and without the vortex pump, was observed only within the dark core of the vortex beam (Fig. 4(e)). These results are in good agreement with the simulations shown in Fig. 4(a).

To confirm such phenomenon in nonlinear absorption of the bilayer graphene, the modulated electric field of the signal beam with and without the vortex pump beam was also measured directly using an electro-optical (EO) sampling method for the enhancement of sensitivity. The signal electric field with the vortex pump beam slightly increased 2–3% of that without the pump beam (defined as modulation depth) around *ω*_0_ (in the ring of the vortex) owing to the saturated absorption, while it exhibits weaker modulation with a depth of <1% around the core of the vortex arising from the unsaturated linear absorption of the graphene (Fig. 4(f)). In fact, the experimental 2D counter map of the modulated spatial form with the vortex pump beam exhibits a slightly off-axial dip around the core of the vortex pump beam (Fig. 4(g)).

The noise-to-signal ratio of the measured linear absorption is limited by the THz vortex intensity. If the THz vortex intensity increases up to 10 times of the saturation intensity *Is*, the intensity modulation depth of the probe beam reaches up to 20%, corresponding to the power density of 7nW/pixel on a THz imager (see Fig. 4(a) and (b)). This value should be enough to develop the super-resolution THz imaging by utilizing a commercial THz imager with a sensitivity of 0.5nW/pixel^36^.

## Conclusions

We have successfully produced a highly intense monocycle THz vortex beam by utilizing a Tsurupica spiral phase plate. The broadband vortex beam centered at 0.6 THz had an average power of 2.3 mW, corresponding to a peak power of 2.3 MW. The topological charge of the THz vortex beam was directly measured to be approximately +1 using an astigmatic focusing technique. We also numerically simulated a spatial form of the THz vortex beam in the near- and far-fields. The simulated THz vortex had an expected value of topological charge of +1.15. There was a good agreement between the experimental and simulation results.

We have further investigated the nonlinear absorption of bilayer graphene by utilizing the pump-probe method with the generated THz vortex beam. The results pave the way for a super-resolution THz imaging system based on nonlinear absorption.

## Methods

### EO sampling for beam profile measurement

Our THz wave generation and detection system was based on a 1-kHz Ti:sapphire regenerative amplifier amplifier (Spitfire Ace, Spectra Physics), delivering 100-fs pulses with a maximum pulse energy of 4 mJ at 800 nm. Intense THz waves were efficiently generated by optical rectification (OR) based on a tilted-pulse-front technique and detected by electro-optic (EO) sampling. The corresponding spectra were obtained by fast Fourier transform (FFT) of the time traces of the measured THz electric fields.

The THz beam profiles were recorded by scanning a pinhole along the beam cross-section. The transmitted THz wave passing through a pinhole was focused onto a 1-mm-thick <110> ZnTe crystal to measure the THz electric field-induced phase retardation of the optical probe pulse in the EO crystal. From the signals measured, we are able to achieve the morphology of the THz beam. Due to lack of suitable methods for sensitive measurement of the THz beam profile, the EO-sampling-based pinhole scan method was employed in the present experiment, whereas one data point through the pinhole acts as one pixel of the THz beam profile. The schematic layout of the system is shown in Fig. 1(a). The high-power THz beam from the prism-cut LiNbO_3_ was divided by using a 6:4 beam splitter to provide THz pump and THz probe pulses. The stronger THz Gaussian pump was converted to THz vortex pump beam after passing a THz spiral phase plate (SPP), while the power of the THz probe beam was substantially attenuated by using two wire-grid polarizers to induce only linear transmission behavior without any nonlinear effects through a bilayer graphene sample. The THz pump and probe beams were spatially and temporally synchronized at the sample position. Lenses with different focal lengths were used to focus the vortex pump and Gaussian probe beams separately for efficient mode matching. The THz vortex pump spatially excited the bilayer graphene and induced nonlinear absorption. After passing through the spatially excited graphene by saturable absorption, the resulting transmitted probe beam was spatially modulated and it exhibited an off-axial shallow dip.

## Additional Information

**How to cite this article**: Miyamoto, K. *et al*. Highly intense monocycle terahertz vortex generation by utilizing a Tsurupica spiral phase plate. *Sci. Rep.* **6**, 38880; doi: 10.1038/srep38880 (2016).

**Publisher's note:** Springer Nature remains neutral with regard to jurisdictional claims in published maps and institutional affiliations.
